# Uptake of a plasticizer (di-n-butyl phthalate) impacts the biochemical and physiological responses of barley

**DOI:** 10.7717/peerj.12859

**Published:** 2022-02-14

**Authors:** Arpna Kumari, Rajinder Kaur

**Affiliations:** 1 Department of Botanical and Environmental Sciences, Guru Nanak Dev University, Amritsar, Punjab, India; 2 Academy of Biology and Biotechnology, Southern Federal University, Rostov-on-Don, Russia

**Keywords:** Di-n-butyl phthalate, Bioaccumulation, Translocation, Morphological responses, Physiological consequences, Oxidative stress

## Abstract

**Background:**

DBP is one of the most commonly used plasticizers for imparting desirable properties to polymers. The introduction of phthalates is reported to have occurred in the late 1920s, and there has been a significant rise in their release into the environment in past decades due to a lack of covalent bonding with the parent matrix. Because of their numerous applications in day-to-day life, phthalates have become ubiquitous and also classified as endocrine disruptors. Hence, several studies have been conducted to investigate the phthalate-mediated toxicities in animals; however, plants have not been explored to the same amount.

**Methods:**

Therefore, in the present study, the accumulation and translocation along with morpho-physiological perturbations in barley plants after 15, 30, 60, and 120 days of exposure to di-n-butyl phthalate (DBP) are investigated using standard protocols.

**Results:**

The maximal accumulation and translocation of DBP in the roots and shoots of barley plants was observed after 60 days of exposure. The exposure of DBP from 15 to 120 days was recorded to decline all the morphological indices (*i.e.,* dry weight, net primary productivity, seed number per spike, and seed weight) of barley plants. The pigments content declined under DBP treatment for all exposure durations except 120 days exposure. Carbohydrate content increased after 15–30 days of exposure afterward it was observed to be decreased under 60 and 120 days of exposure. The protein content was declined in DBP stressed plants for 15–120 days. Proline content was increased in all exposure durations and maximal percent increase was recorded in 120 days of exposure. MDA content showed an increase at earlier exposure durations then followed by a decline in long-term exposure. Hydrogen peroxide content increased at all exposure durations. There were significant alterations observed in the activities of all antioxidative enzymes in comparison to the control. Furthermore, DBP stressed plants after 60 days were analyzed for the macromolecular variations using Fourier transform infrared spectroscopy (FTIR).

**Conclusion:**

Thus, the outcomes of the current work provide an appraisal of phthalates’ uptake and translocation mediated phytotoxic responses in barley plants. These observations can help in developing genetically modified edible plants that are resistant to phthalates uptake, thereby ensuring food security.

## Introduction

Phthalates are well-avowed plasticizers and additives that are commonly used in fabrics, food containers, pharmaceuticals, medical devices, polyvinyl chloride, detergents, and personal care items (Zhang et al., 2020). However, because of their pervasiveness, they are classified as a high-risk group of emerging contaminants. Phthalates are weakly or physically bonded to the parent polymer, thereby they can be released into the environment during plastic manufacturing, storage, use, and processing (Giuliani et al., 2020). In different components of environment, the persistence of phthalates has been reported from one day to several months or longer (Staples et al. 1997). Soil serves as a significant sink for phthalates in the terrestrial ecosystem, which leads to their accumulation in edible plants, raising food security concerns because they have been identified as potential endocrine disruptors (Kumari & Kaur, 2020b).

On the other hand, to enhance or sustain agricultural production due to elevated demands and global climate change, plasticulture has emerged as a promising practice. Thus, plastic film mulching gained great popularity and also reported as a successful technique especially for arid and semi-arid areas (Shi et al., 2019). For example, the application of plastic film mulching has been considerably intensified from 6,000 tons to 1.2 million tons during 1982-2011 (He et al., 2018). Undoubtedly, plastic mulching is reported to increase soil warmth, reduces weeds and insect pests, maintains soil moisture, increases crop yields, and efficiently uses nutrients, *etc*. (Kumari & Kaur, 2021). But, due to the inclusion of high amounts of additives (*e.g.*, phthalates that can up to 10–60% by weight), the usage of plastic film mulching has sparked a lot of concern in the previous decade (Bergé et al. 2013). Another concerning issue is the long-term persistence of plastic film residues in agricultural fields, which can endure up to 400 years in natural conditions and continue to emit phthalates into the fields (Xu et al., 2005). The other sources of phthalate contamination in arable soils are plastic garbage incineration, plasticizers volatilization from plastics/polymers, industrial dust sedimentation, use of sewage sludge or biosolids, use of reclaimed water for irrigation, chemical and organic fertilizers, pesticides, leaching from plastic waste, paint spraying, *etc*. (Kumari & Kaur, 2020b). On the other hand, food quality and safety, are crucial factors that must not be overlooked because they are intricately intertwined with human health. Substantiating to several reports, phthalates are documented to accumulate up to hazardous levels in edible plants that are responsible to raise food safety concerns globally (Campanale et al. 2020). Furthermore, according to some studies, the total amount of accumulated phthalates in plants was substantially higher than the maximum permissible limit set by the California Office of Environmental Health Hazard Assessment. In plants, phthalates’ accumulation is reported to affect the normal metabolic process that is apparent in the form of morpho-physiological consequences (Kumari & Kaur, 2020b).

With this background, the current work was designed to distinctly envisage the uptake, translocation of one of the most frequently used phthalates (*i.e.,* DBP) along with subsequent phytotoxic responses in barley plants grown in pots under natural conditions. Barley is an important cereal crop of the world since it has not been well explored for the accumulation and translocation of DBP along with its perturbations. In this work, the treatment concentrations of DBP were kept high due to the prevalence of phthalates in terrestrial ecosystems. Plus, the selected doses were also based on the inhibitory concentrations of DBP that were calculated using Probit analysis. Therefore, the current study was designed that aimed to investigate (i) uptake and translocation of DBP in barley; (ii) DBP induced plant growth and yield responses; (iii) pigments, osmolytes (soluble sugars, proteins, and proline), stress metabolites (malondialdehyde and hydrogen peroxide) analyses under DBP stress; (iv) DBP mediated modulations in the antioxidative defense system of barley, (v) FTIR analysis to observe macromolecular variations.

## Material and Methods

The seeds (*Hordeum vulgare* var. VLB-118) were purchased from the Hill Agricultural Research and Extension Centre, H.P., India and DBP (CAS: 84-74-2, purity: 99%) was purchased from Himedia Laboratories Private Limited (India). The other chemicals used were of analytical grade.

### Soil preparation, spiking, and pot experiment

The soil used in the present study was collected from the uncontaminated site (0–20 cm depth) of Guru Nanak Dev University, Amritsar (India). The soil was air-dried, sieved using a sieve (2 mm), and analyzed for physico-chemical characteristics using standard methods (Table S1). After physico-chemical analysis of soil, it was spiked with DBP and concentrations were set at 0, 800, 1600, and 2400 mg/kg. The DBP spiking was done according to the method of Cai et al. (2017). Briefly, the soil was spiked with DBP, mixed thoroughly, and left for 48 h for acetone evaporation. 8 kg soil was kept in each ceramic pot (25  ×18 cm, internal diameter × height).

### Harvesting of barley plants

The plants were harvested after 15, 30, 60, and 120 days of exposure with DBP. The plants were washed under running tap water followed by distilled water for the removal of dust and soil particles, dried in the paper towel, and stored at −80 °C till analyses.

### Determination of accumulation and translocation of DBP

For extraction of DBP from shoots, roots and seeds, the sample was homogenized and extracted with acetone and then with hexane in an ultrasonic bath as per the method of Kumari, Arora & Kaur (2020c). For the determination of DBP concentration in soil, the sample was extracted in dichloromethane for 6 h. The obtained extract of plant and soil sample was dissolved in acetonitrile, filtered through 0.22 µm membrane filter, and DBP analysis was carried out using the RP-UHPLC-PDA method of Kumari, Arora & Kaur (2020c). The amount of DBP in different parts of barley was used for the determination of shoot accumulation factor (SAF), root accumulation factor (RAF), and seed accumulation factor (SdAF). Translocation factor (TF) from root to shoot (R:S) and shoot to seed (S:Sd) was also calculated. For the calculation of accumulation and translocation factors, equations are given by Li et al. (2014) were used:

(1) \begin{eqnarray*}RAF= \frac{\text{Phthalate concentration in roots}}{\text{Phthalate concentration in soil}} \end{eqnarray*}

(2) \begin{eqnarray*}SAF= \frac{\text{Phthalate concentration in shoots}}{\text{Phthalate concentration in soil}} \end{eqnarray*}

(3) \begin{eqnarray*}SdAF= \frac{\text{Phthalate concentration in seeds}}{\text{Phthalate concentration in soil}} \end{eqnarray*}

(4) \begin{eqnarray*}T{F}_{\mathrm{(R:S)}}= \frac{\text{Phthalate concentration in shoots}}{\text{Phthalate concentration in roots}} \end{eqnarray*}

(5) \begin{eqnarray*}T{F}_{\mathrm{(S:Sd)}}= \frac{\text{Phthalate concentration in seeds}}{\text{Phthalate concentration in shoots}} \end{eqnarray*}

### Morphological indices

After the harvesting of plants, the dry weight was calculated using the method of Lin et al. (2012). To know dry weight accumulation, net primary productivity was observed using the method given by Malik (2009). Moreover, the seeds number per spike and dry weight of 100 seeds were also recorded.

### Biochemical parameters

The plants after 15, 30, 60, and 120 days treatment were analyzed for different biochemical parameters as follows:

#### Pigments content

Plant samples were analyzed for total chlorophyll (chl) and carotenoids content using Arnon (1949) method. The samples were extracted using acetone (80%) and absorbance was recorded at 470, 645, and 663 nm. The calculations of total chlorophyll and carotenoids contents were done using the equations of Arnon (1949) and Lichtenthaler & Wellburn (1983) respectively.

#### Carbohydrate content

The Anthrone reagent method was employed for the determination of carbohydrate content (Yemm & Willis, 1954). The untreated and treated samples were acid hydrolyzed (2.5 N HCl) followed by neutralization using sodium carbonate. The volume of the solution was raised to 100 mL that was later centrifuged. The supernatant was collected and to that Anthrone reagent was added. The reaction mixture boiled, cooled to ambient temperature and then absorbance was observed at 630 nm. The glucose was used as a standard.

#### Protein content

The protein content of plant samples after 15, 30, 60, and 120 days of treatment with DBP was analyzed using the Bradford method (1976). The homogenate of samples was prepared in potassium phosphate buffer (PPB) that was centrifuged at 12,000 rpm for 20 min and the temperature was set at 4 °C. After the collection of supernatant, the Bradford reagent was added and absorbance was recorded at 595 nm. Bovine serum albumin was used as a standard.

#### Proline content

For the estimation of proline content, the method given by Bates, Waldren & Teare (1973) was used. The plants homogenate was prepared using sulfosalicylic acid (3%, w/v) and then filtered. To the filtrate, acid ninhydrin and glacial acetic acid were added that were followed by heating at 100 °C for 1 h. To terminate the reaction, test tubes were placed into an ice bath. To this solution, toluene was added and mixed vigorously, and absorbance was recorded at 520 nm. L-proline was used as a standard.

#### Malondialdehyde (MDA) content

The malondialdehyde content of barley plants was determined using Heath & Packer (1968) method. The plant samples were homogenized using trichloroacetic acid (TCA). The homogenate was centrifuged at 10,000 rpm for 5 min and thiobarbituric acid solution (prepared in 20% TCA) was added to the supernatant. This reaction mixture was heated at 95 °C for half an hour and after cooling, the absorbance was observed at 532 and 600 nm.

#### Hydrogen peroxide (H_**2**_O_**2**_) content

The method of Alexieva et al. (2001) was used to determine the H_2_O_2_ content in barley plants treated for different durations. The samples were extracted using TCA (0.1%). To the extract, potassium phosphate buffer and potassium iodide were added. Then, the reaction mixture was kept in dark for 1 h and absorbance was recorded at 390 nm. H_2_O_2_ was used as a standard.

### Antioxidant enzymatic activities

The plants treated for 15, 30, 60, and 120 days were analyzed to know DBP induced effect on the activities of antioxidant enzymes.

#### Superoxide dismutase (SOD) activity

The method proposed by Kono, Takahashi & Asada (1979) was used to determine the SOD activity in DBP stressed plants. SOD activity was observed as a decline in absorbance that was recorded at 540 nm.

#### Guaiacol peroxidase (POD) activity

POD activity was analyzed using the method of Pütter (1974). For the preparation of the reaction mixture, PPB, guaiacol, hydrogen peroxide, and enzyme extract were used in the desired proportion. The absorbance was measured at 436 nm and for the determination of enzymatic activity, the extinction coefficient (25.5 mM^−1^cm^1^) was used.

#### Catalase (CAT) activity

For evaluation of CAT activity, the protocol of Aebi (1984) was used. In brief, the reaction mixture was prepared by using PPB, 150 mM of hydrogen peroxide, and enzyme extract. The value of the extinction coefficient *i.e*., 39.4 mM^−1^cm^−1^ was used for the calculation of CAT activity.

#### Ascorbate peroxidase (APX) activity

The reaction solution for determining APX activity consisted of PPB, ascorbate, hydrogen peroxide, and enzyme extract. The absorbance was recorded at 290 nm and the extinction coefficient (2.8 mM^−1^cm^−1^) was employed to calculate the APX activity (Nakano & Asada, 1981).

#### Glutathione reductase (GR) activity

For estimation of GR activity, the standard protocol of Carlberg & Mannervik (1985) was used. The absorbance of the reaction mixture was read at 340 nm. The calculation was performed using the value of extinction coefficient (*i.e*., 6.22 mM^−1^cm^−1^).

### FTIR analysis

FTIR analysis of the plant sample was done using Cary 630 FTIR (Agilent Technologies). The plant samples from different durations were oven-dried at 80 °C for 48 h. The oven-dried plant samples were pulverized using pestle mortar. For the FTIR analysis, the pulverized sample was mixed with potassium bromide (KBr) followed by pellets formation using agate mortars. The absorbance spectrum was obtained for a range between 400 and 4000 cm^−1^.

### Statistical analyses

All the experiments were performed in triplicate. The mean and standard error of data was calculated, and analysis was carried out using one- and two-way analysis of variance (ANOVA). The differences (*p* ≤ 0.05, 0.01) among the means were compared by an honestly significant difference (HSD) using Tukey’s multiple comparison test. Pearson’s bivariate correlation analysis and principal component analysis (PCA) were performed using SPSS 22.0 version (SPPS Inc., Chicago, IL).

## Results

### Accumulation of phthalates

**Figure 1:**
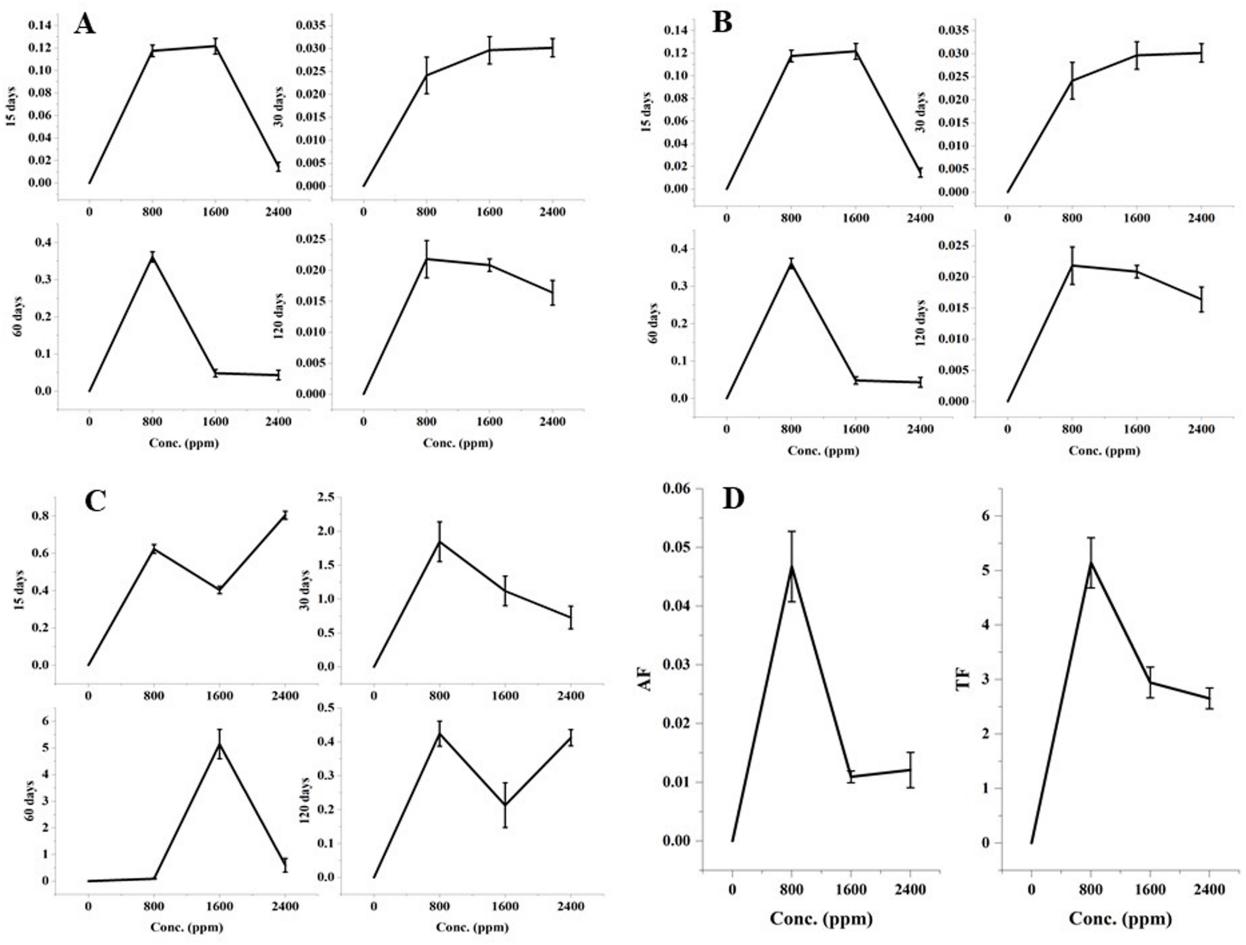
(A) RAF after 15–120 days treatment, (B) SAF after 15–120 days treatment, (C) TF (root:shoot) after 15–120 days treatment, (D) AF (shoot:seed) and TF (shoot:seed) after 120 days treatment with DBP.

The significant variations in the accumulation and translocation of DBP in barley plants were observed as compared to the control after 15, 30, 60, and 120 days of treatment with DBP (Fig. 1).

#### Root accumulation factor

Root accumulation factor showed a significant (*p* ≤ 0.01) alterations with an increase in concentration for 15-120 days exposure to DBP. The maximal values of the root accumulation factor were recorded in case of 60 days exposure (0.361) followed by 15 days, 30 days, and 120 days exposure.

#### Shoot accumulation factor

There were significant (*p* ≤ 0.01) dose and time-dependent variations in the shoot accumulation factor. The maximal shoot accumulation factor value was recorded at 1600 ppm DBP treatment for 60 days.

#### Seed accumulation factor

The seed accumulation factor was observed after 120 days of treatment. The significant (*p* ≤ 0.01) alterations in the seed accumulation factor were observed with an increase in the doses of DBP. The maximum value for the seed accumulation factor was 0.047 which was found at 800 ppm DBP.

#### Translocation of phthalates

#### Root to shoot

Alike accumulation factors, there were significant (*p* ≤ 0.05, 0.01) variations in the values of the DBP translocation factor under different exposure durations. The maximal value of the translocation factor from root to shoot was recorded at 1600 ppm under 60 days of exposure to DBP.

#### Shoot to seed

The significant (*p* ≤ 0.05, 0.01) translocation of DBP from shoots to seeds was observed. The values of translocation factor from shoot to seed were 5.14, 2.95, 2.65 at 800, 1600, 2400 ppm, respectively.

### Effect of DBP on growth

The effects of DBP on different growth parameters are presented in Table 1 along with their ANOVA summary.

**Table 1:** Effect of DBP on dry weight, net primary, seed number per spike, and seed weight productivity after 15, 30, 60, and 120 days exposure.

Conc. (ppm)	Dry weight (g)	NPP
**15 DAT**		
**0**	0.032 ± 0.002	0.0021 ± 0.0001
**800**	0.025 ± 0.001	0.0017 ± 0.0001
**1600**	0.032 ± 0.000	0.0021 ± 0.0001
**2400**	0.031 ± 0.001	0.0021 ± 0.0001
**ANOVA summary**		
**HSD**	0.006	0.0004
**F-ratio**	6.20^*^	6.20^*^
**30 DAT**		
**0**	0.1022 ± 0.0087	0.0034 ± 0.0003
**800**	0.0445 ± 0.0032	0.0015 ± 0.0001
**1600**	0.0481 ± 0.0113	0.0016 ± 0.0004
**2400**	0.0531 ± 0.0190	0.0018 ± 0.0006
**ANOVA summary**		
**HSD**	0.054	0.0018
**F-ratio**	5.09^*^	5.09^*^
**60 DAT**		
**0**	0.7344 ± 0.2586	0.0122 ± 0.0043
**800**	0.2158 ± 0.0653	0.0036 ± 0.0011
**1600**	0.0972 ± 0.0212	0.0016 ± 0.0004
**2400**	0.0649 ± 0.0072	0.0011 ± 0.0001
**ANOVA summary**		
**HSD**	0.60	0.0101
**F-ratio**	5.40^*^	5.40^*^
**120 DAT**		
**0**	1.6216 ± 0.8854	0.0149 ± 0.0025
**800**	0.4552 ± 0.1289	0.0038 ± 0.0011
**1600**	0.2173 ± 0.0215	0.0018 ± 0.0002
**2400**	0.1487 ± 0.0163	0.0012 ± 0.0001
**ANOVA summary**		
**HSD**	0.7482	0.0062
**F-ratio**	21.65^**^	21.65^**^
**Yield parameters**	**Seed number per spike**	**Seed weight (g) 100 seeds**
**0**	28.89 ± 0.40	6.36 ± 0.45
**800**	14.44 ± 0.31	4.10 ± 0.35
**1600**	11.53 ± 0.33	3.75 ± 0.51
**2400**	7.00 ± 0.28	1.54 ± 0.20
**ANOVA summary**		
**HSD**	53.09^**^	24.79^**^
**F-ratio**	4.73	1.46

**Notes.**

Data presented as means ± S.E.

** means significant at *p* ≤ 0.01.

* *p* ≤ 0.05.

#### Effect on plants dry weight

DBP exposure significantly (*p* ≤ 0.05, 0.01) declined the dry weight of plants in all exposure periods. The maximal percent decline was recorded after 120 days of treatment (*i.e*., 92%). In 15 days of treatment, a slight decline in the dry weight was observed *i.e.,* 21%, whereas, in 30 days of exposure, it was more than 50% in comparison to the control.

#### Effect on net primary productivity

The net primary productivity (NPP) of barley declined significantly (*p* ≤ 0.05, 0.01) under the stress of DBP for 15, 30, 60, and 120 days. The trend of decline was similar to that of dry weight.

#### Effect on crop yield

The exposure of DBP significantly (*p* ≤ 0.01) declined the seeds number per spike and 100 seeds weight. The percent decline was 50, 60, 76% at 800, 1600, 2400 ppm respectively for seed number. Similarly, in the case of seed weight, the percent decline was 36, 41, 76% at 800, 1600, 2400 ppm respectively as compared to the control.

### Effect of DBP on physiological indices

**Figure 2:**
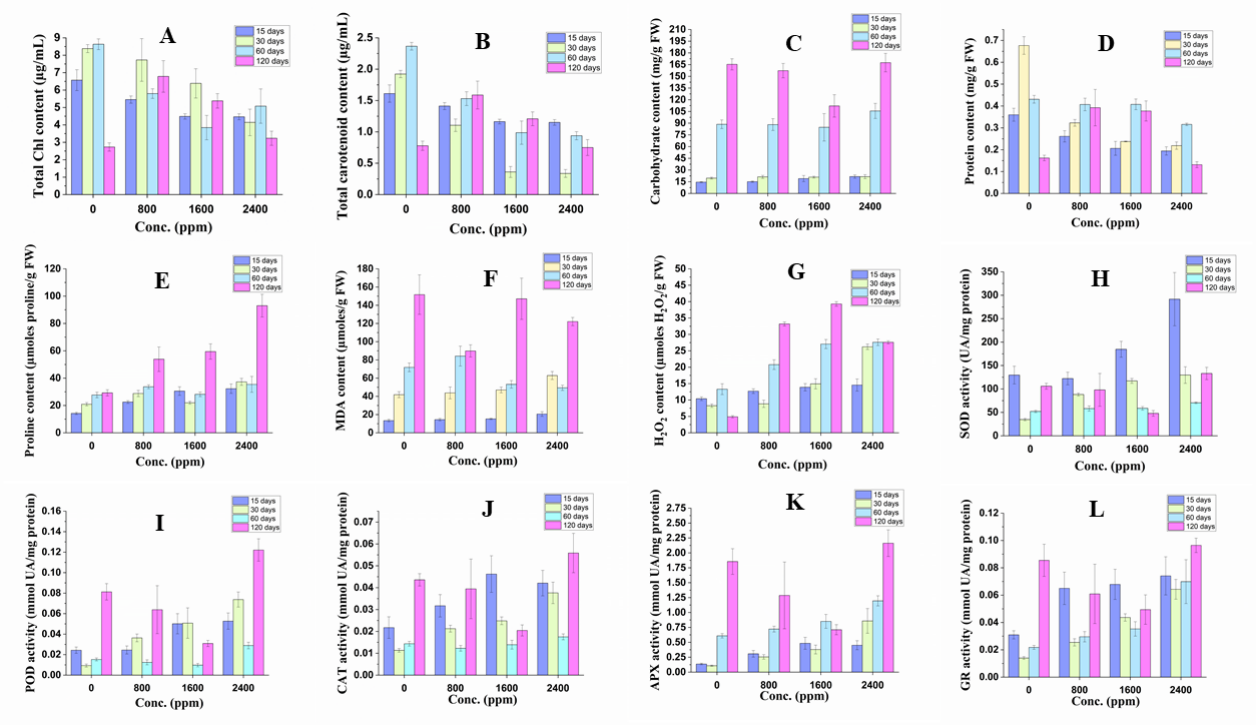
Effect of DBP on the contents of (A) total Chl, (B) total carotenoid, (C) arbohydrate, (D) protein, (E) proline, (F) MDA, (G) H_2_*O*_2_; (H) SOD activity, (I) POD activity, (J) CAT activity, (K) APX activity, (L) GR activity in barley. Results are expressed as mean ± S. E.; *n* = 9.

The plant under DBP stress showed significant alterations in physiological indices in comparison to the control as shown in Fig. 2 and Table S2.

#### Pigments content

The content of total Chl decreased significantly (*p* ≤ 0.01) in all treatment durations (17 to 32% in 15 days; 8 to 51% in 30 days; 33 to 55% in 60 days) except in 120 days treatment, where it was found to be increased that ranged from 18 to 148%. The content of carotenoids followed similar trends of a significant decrease or increase with DBP treatment.

#### Carbohydrate content

The content of carbohydrates increased significantly (*p* ≤ 0.01) in 15 and 30 days of exposure to DBP and the percent increase was recorded to range from 4 to 49% and 7 to 10% respectively, whereas it decreased initially at 800 and 1600 ppm then increased at 2400 ppm after 60 and 120 days of exposure to DBP.

#### Protein content

The content of protein was decreased significantly (*p* ≤ 0.01) in 15, 30, and 60 days of exposure. The percent decrease was 27, 43, 46% (15 days), 52, 65, 68% (30 days) and 6, 5, 27% (60 days) at 800, 1600, 2400 ppm, respectively as compared to the control. After 120 days of treatment with DBP, the protein content increased significantly at 800 and 1600 ppm and then decreased by 19% in comparison to the control.

#### Proline content

There was a significant (*p* ≤ 0.01) increase in the content of proline in barley plants under all exposure durations. The percent increase in 15, 30, 60, and 120 days of exposure ranged from 59 to 127%, 38 to 79%, 2 to 28%, and 84 to 218%, respectively as compared to the control.

#### MDA content

The content of MDA was increased significantly (*p* ≤ 0.01) in 15 and 30 days of treatment with DBP and the percent increase ranged from 8 to 54% and 5 to 50%, respectively. On the other hand, under 60 and 120 days of exposure to DBP, the MDA content was decreased, and the maximum percent decrease was 31% and 41%, respectively as compared to the control.

#### H_**2**_O_**2**_ content

The content of H_2_O_2_ was increased significantly (*p* ≤ 0.01) under all the treatment durations. The percent increase was 22, 34, 40% (for 15 days), 7, 81, 217% (for 30 days), 56, 103, 107% (for 60 days), and 578, 703, 464% (for 120 days) at 800, 1600, 2400 ppm, respectively as compared to the control. The trend of increase in H_2_O_2_ content was almost dose and duration dependent.

### Effect on antioxidative enzymes activities

The response of antioxidative enzymes’ activities was recorded to be varied significantly in barley plants treated with DBP for 15–120 days (Fig. 2 and Table S2).

#### SOD activity

After 15 days of treatment with DBP, the SOD activity decreased initially followed by a significant increase at 1600 and 2400 ppm by 43 and 125% as compared to the control. There was a significant increase in SOD activity after 30 and 60 days of treatment and the percent increase was 154, 238, 274% and 12, 13, 35% at 800, 1600, 2400 ppm, respectively as compared to control respectively. Under DBP stress for 120 days, the SOD activity was decreased at 800 and 1600 ppm then followed by an increase at 2400 ppm.

#### POD activity

After 15 and 30 days of exposure to DBP, the activity of POD significantly (*p* ≤ 0.01) increased with increase in concentrations and the percent increase was 1, 106, 117 and 289, 444, 690% at 800, 1600, 2400, respectively as compared to the control. The POD activity was recorded to decline significantly at 800–1600 ppm then followed by an increase at 2400 ppm after DBP treatment for 60 and 120 days.

#### CAT activity

CAT activity followed similar trends of significant (*p* ≤ 0.01) variations as recorded in the case of POD activity in all exposure durations. After 15 and 30 days of treatment, the activity was observed to increase significantly, and the percent increase ranged from 46 to 113% and 88 to 232% respectively, in comparison to the control. Whereas, after 60 and 120 days of exposure to DBP, CAT activity declined significantly which was followed by an increase at 2400 ppm.

#### APX activity

APX activity increased significantly (*p* ≤ 0.01) in all exposure periods except at 800 and 1600 ppm after 120 days of treatment. The percent increase was 126, 25, 231% (15 day); 137, 252, 698% (30 days), 19, 40, 96% (60 days) at 800, 1600, 2400 ppm, respectively as compared to the control, while the percent increase in DBP stressed plants for 120 days at 2400 ppm was 31%.

#### GR activity

GR activity followed a similar significant (*p* ≤ 0.05, 0.01) trend as recorded in case of APX activity. After 15, 30, and 60 days of exposure to DBP, GR activity increased and the percent increase ranged from 110–130%, 82–359%; 36–221% for 15, 30, and 60 days of exposure, respectively. While, in barley plants treated for 120 days, the increase in GR activity was only observed at 2400 ppm (*i.e.,* 13%).

### FTIR observations

**Figure 3:**
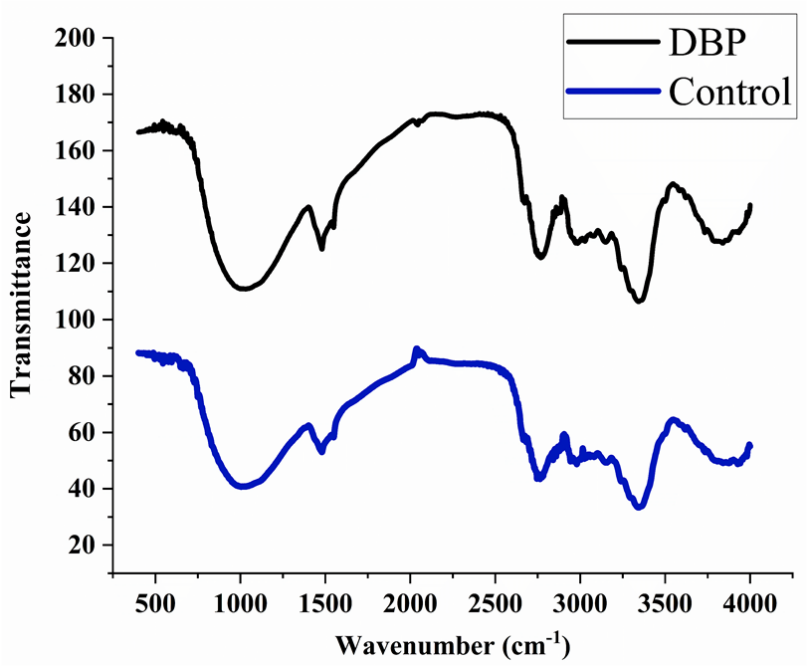
FTIR spectra for (A) control, (B) DBP treated barley plants after 60 days exposure.

The peaks were observed at 3395, 2920, 2350, 1735, 1718, 1654, 1638, 1541, 1522, 1457, 1357, 1246, 1056, 782, 665, and 559 cm^−1^ in the control sample after 60 days of exposure. The treated samples with DBP showed the peaks at 3369, 2920, 2851, 2357, 1727, 1632, 1414, 1250, 1157, 1056, 816, 779, 669, and 562 cm^−1^ (Fig. 3).

### PCA analysis

**Figure 4:**
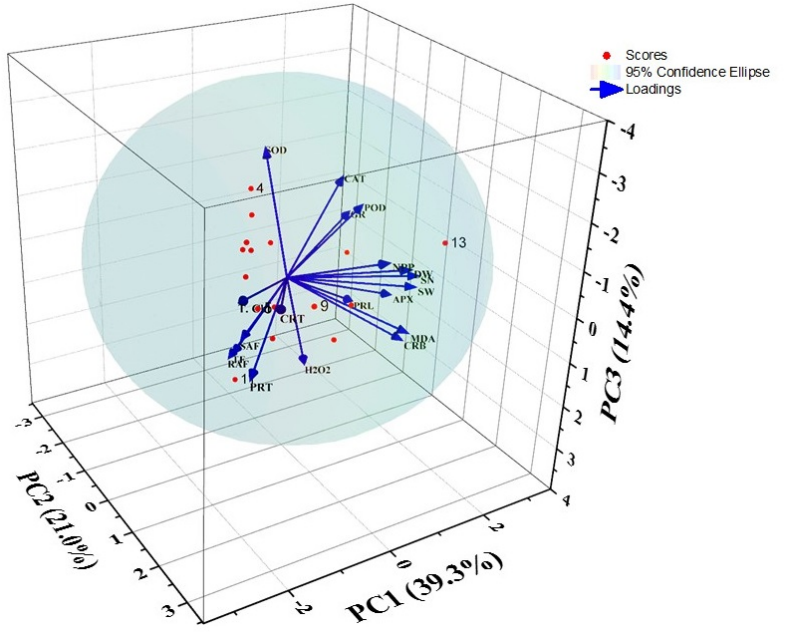
Biplot of the PCA for different indices of barley under DBP stress.

After PCA analysis, four factors were extracted that provided 84.33% variations. The principal components *i.e.,* PC1, PC2, PC3, and PC4 showed 39.30, 20.97, 14.40, and 9.68% of the variance respectively (Fig. 4 and Table 2). The factors loadings were categorized into three classes as strong (>0.75), moderate (0.75−0.50), and weak (<0.50) (Liu et al., 2003). Thus, PC1 showed strong positive loadings with SOD, POD, CAT, and GR activities, while strong negative loadings with total chlorophyll content. It also exhibited moderate negative loadings with carotenoids content. PC2 showed strong positive loadings with dry weight, NPP, MDA content, seed weight, and seed number as well as there were moderate positive loadings with carbohydrate content and APX activity. PC3 had PC3 had moderate positive loadings with carbohydrate content, MDA content, and APX activity. Plus, PC3 showed only strong positive loadings with H_2_O_2_ and proline contents. The principal component 4 showed strong positive loadings with SAF and TF.

**Table 2:** Factor loading values, eigenvalues, % variance and % cumulative for the four factors extracted from the principal component analysis.

Indices	Principle component			
	PC1	PC2	PC3	PC4
DW	0.054	**0.967**	−0.181	−0.131
NPP	−0.129	**0.844**	−0.340	−0.215
TChl	**−0.792**	−0.254	−0.187	−0.369
CRT	**−0.726**	0.033	−0.139	−0.326
CRB	0.091	**0.722**	**0.622**	0.007
PRT	**−0.926**	−0.076	0.016	0.009
PRL	0.349	0.125	**0.861**	−0.170
MDA	0.044	**0.753**	**0.566**	−0.085
H_2_O_2_	0.019	−0.082	**0.888**	0.210
SOD	**0.760**	−0.307	−0.316	−0.196
POD	**0.802**	0.249	0.320	−0.293
CAT	**0.885**	0.133	0.113	−0.294
APX	0.469	**0.605**	**0.545**	0.032
GR	**0.877**	0.217	0.268	−0.097
RAF	−0.115	−0.219	0.132	0.494
SAF	−0.016	−0.111	−0.065	**0.950**
TF	0.020	−0.113	−0.025	**0.907**
SW	0.180	**0.841**	0.262	−0.179
SN	0.240	**0.889**	0.166	−0.161
**Eigen values**	7.466	3.984	2.735	1.838
**%Variance**	39.295	20.968	14.396	9.675
**Cumulative%**	39.295	60.264	74.659	84.334

**Notes.**

Here, DW, dry weight; NPP, net primary productivity; TChl, total chlorophyll; CRT, carotenoids; CRB, carbohydrates; PRT, proteins; PRL, proline; MDA, malonaldehyde; H2O2, hydrogen peroxide; SOD, superoxide dismutase; POD, guaiacol peroxidase; CAT, catalase; APX, ascorbate peroxidase; SAF, shoot accumulation factor; RAF, root accumulation factor; TF, translocation factor; SW, seed weight; SN, seed number. Black bold indicates strong loadings, red bold colour indicates moderate loadings and plain black is form weak loadings

### Correlation analysis

**Figure 5:**
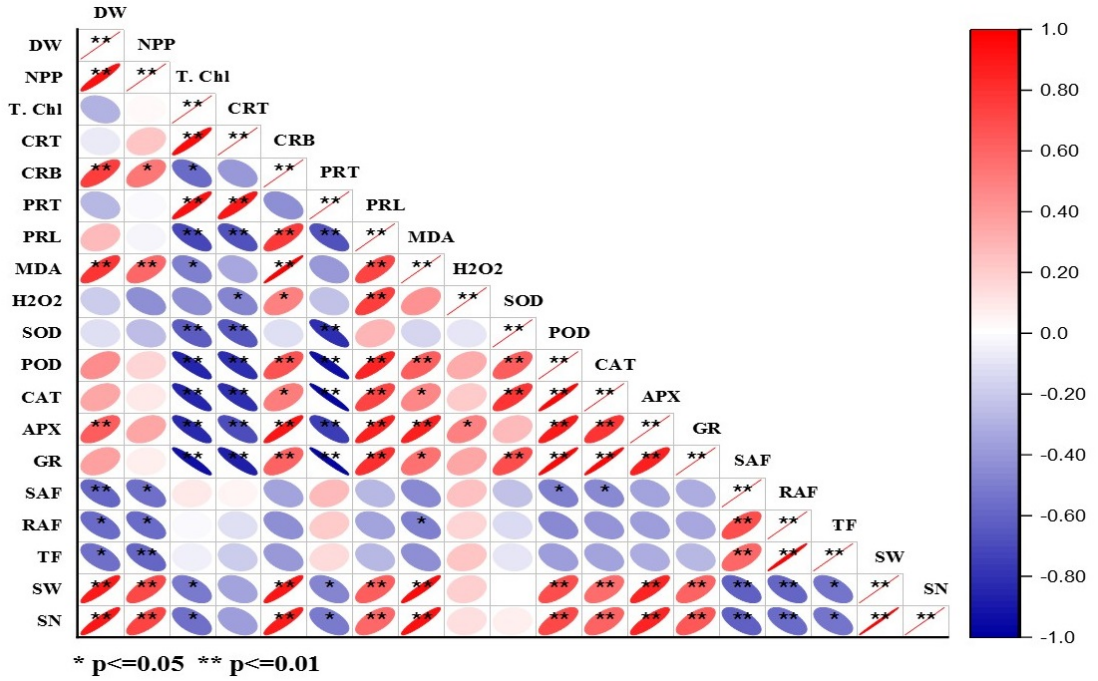
Heatmap of Pearson’s correlation coefficient matrix for different parameter of barley under DBP stress.

The correlation matrix for DBP induced morpho-physiological, accumulation, and translocation factors are presented in Fig. 5 and Table S2. The dry weight of plants showed positive correlations with NPP, carbohydrate content, MDA content, APX activity, seed weight, and seed number. Whereas, NPP had a positive correlation with seed weight and seed number that means net primary productivity and yield of the crop are interlinked. Total chlorophyll exhibited a positive correlation with total carotenoid content and protein content, while it had a negative correlation with the activities of POD, CAT, APX, and GR (at *p* < 0.05, 0.01). The alike pattern of correlation was observed in the case of total carotenoids to that of total chlorophyll content. There was a positive correlation of proline content, MDA content, APX activity, seed weight, and seed number with carbohydrate content at *p* < 0.01. In the case of protein content, there was a negative correlation with all antioxidative enzymes except APX (at *p* < 0.01). The proline content had a significant (at *p* < 0.05, 0.01) positive correlation with MDA content, H_2_O_2_ content, POD, CAT, APX, and GR activities. The MDA content showed a significant positive correlation (at *p* < 0.05, 0.01) with APX, seed weight, and seed number. SOD activity is positively correlated with CAT and GR activities at *p* < 0.05, 0.01. Similarly, POD activity exhibited a significant positive correlation with CAT, APX, and GR activities. CAT activity had a significant positive correlation with APX and GR activities. APX activity showed the positive correlation with GR activity with GR activity, seed weight, and seed number at *p* < 0.05, 0.01. Shoot accumulation factor showed a significant positive correlation with translocation factors. Likewise, seed weight and seed number are positively correlated.

## Discussion

The values of accumulation factor were observed to be varied significantly at different growth stages of barley plants. With an increase in DBP concentrations, the values of RAF and SAF were observed to decrease considerably. The transport of phthalate from roots to aboveground tissue was expressed in terms of translocation factor (TF) and the values of T *F*_(R:S)_ were also recorded to vary significantly with dose and treatment durations. The variations in the values of phthalates’ accumulation factors were also recorded in some works. In a study, the values of accumulation factor (AF) was found to range from 5.8 to 17.9 in plants that were grown in the vicinity of electronic waste recycling site (Ma et al., 2013a). Likewise, higher values of phthalates’ AF were observed in wheat and maize (Tan et al. 2018). In another study, AF was calculated for six phthalates in the grains of *Triticum aestivum* and *Zea mays* that were observed to vary from 0.33 to 33.75 (Li et al., 2018b). DBP has been reported to accumulate and translocate like other lipophilic pollutants. In the rhizosphere, it gets enriched at the root surface and enters *via* roots along with water through the cuticle-free unsuberized cell wall (Müller & Kördel, 1993; Kvesitadze et al., 2006; Zhang et al., 2017). The cell wall between the cells of the root cortex is porous, thereby it can move freely before it reaches the endodermis (Trapp & Mc Farlane, 1994). DBP is reported to be translocated to several plant organs after accumulating in plants’ roots. Also, phthalates’ uptake has been shown to be influenced by solubility, contaminant molecular mass, pH, temperature, growth phase, *etc*. (Korte et al. 2000). Thus, these factors might have impacted the translocation of DBP at different exposure periods in current work. Moreover, the previous reports have revealed the accumulation of phthalates in vegetables and cereal plants and also their translocation from root to shoot (stem, leaves, and seeds) (Cai et al., 2008a; Zhao et al., 2015). Thus, the current observations of accumulation and translocation of DBP in barley exhibited considerable coherence with the existing literature.

DBP stress declined the dry weight of barley plants after all exposure durations. The biomass of different plants was declined under the exposure of DBP (Dueck et al., 2003). Similarly, the fresh weight was declined in DBP treated *Brassica rapa* subsp*. chinensis* (Liao, Yen & Wang, 2006). The growth and biomass also declined by DBP exposure in *Ipomoea aquatica* (Cai et al., 2008c; Chen et al., 2011). The wheat under the exposure of DBP showed a decrease in the dry weight (Gao et al., 2016). Due to the adverse effects of phthalates on germination as reported in several studies, the growth and development of barley plants might be affected because seed germination determines the subsequent growth quality of the plant (Gao et al., 2021). Thus, a reduction in biomass of barley plants could be caused by DBP accumulation that disturbed the normal seed germination and subsequent developmental processes.

Under the exposure of DBP, plants grown for different durations showed a significant decline in NPP. The decline in NPP may be due to the intrusions caused by the exposure of DBP in barley plants. In the present study, the exposure of DBP caused a significant reduction in the number of seeds per spike and seeds weight as compared to the control. The decline in the yield of barley was observed to be associated with seed size and number under early and late drought (Jamieson, Martin & Francis, 1995). Under abiotic stresses, the reproductive development of plants is reported to be highly vulnerable. The stress can delay or inhibit flowering by affecting floral induction and development. Moreover, meiosis is also reported to be affected which leads to pollen sterility in plants. The studies on crops like *Triticum aestivum*, *Oryza sativa,* and *Sorghum bicolor* also revealed that the stamen or carpel primordia arrest causes the reduction in fertility (Bommert et al., 2005; Yoshida & Nagato, 2011). Subsequently, reduced flower fertility leads to a loss in crop yield. Thus, DBP may have disrupted the floral induction as well as the development of barley, resulting in sterility and a reduction in crop production.

The content of pigments was found to be decreased in barley plants treated with DBP for 15, 30, and 60 days, while after 120 days of exposure, plants showed an increase in total chlorophyll content. There was a significant dose and time-dependent decline in the content of chlorophyll of *Brassica rapa* var. *chinensis* under DBP stress (Liao, Yen & Wang, 2009). The decline in the content of chlorophyll was recorded DBP stressed cucumber seedlings (Zhang et al., 2015). The exposure of DBP for 7 days showed similar trends of variation in the contents of pigment in barley seedlings (Kaur et al. 2017). Another plasticizer *i.e.,* BPA also enhanced the content of pigments in *Arabidopsis thaliana* (Rapala, Plucinski & Jedynak, 2017). Similarly, in another study, BPA increased the activity of those enzymes involved in chlorophyll biosynthesis (Jiao et al., 2017). Therefore, an increase in pigments content after 120 days might be a part of strategies to combat DBP induced stress. However, the diminution in the contents of total chlorophyll for other exposure durations might have occurred due to thylakoid and chlorophyll degradation mediated by DBP exposure. Further, a decline in chlorophyll contents could also be due to increased chlorophyllase activity along with chloroplast degradation and chlorophyll oxidation caused by ROS (Parida, Das & Das, 2002).

The total carotenoid content also declined in 15, 30, 60 days of exposure to DBP and the plants treated for 120 days with DBP showed an increase in total carotenoids. The increase in carotenoid content is observed under various environmental stresses. The carotenoids are referred to as accessory pigments that are also known as low-molecular-weight antioxidants as reported to involve in the diminution of damaging effects of singlet oxygen (^1^O_2_) and peroxyl radicals. Moreover, the oxidation products of *β*-carotene are observed to accumulate in the plant under stressed conditions as a combat strategy (Uzilday et al., 2015). Therefore, an increase in the content of total carotenoid in barley plants after 120 days might be a part of the defense mechanisms of plants. Whereas a decline in total carotenoids under other exposure durations may be due to the loss of their protective functions against DBP.

In DBP stressed plants, there was an almost increase in the content of carbohydrates up to 120 days of exposure. The observations of the present study for carbohydrate content were coherent to previous studies. The content of total soluble sugars was enhanced significantly under the exposure of DBP in rape seedlings (Ma et al., 2013b). The increase in carbohydrate content was observed by Li et al. (2006) in *Potamogeton maachianus* with the increase in DBP concentrations. In mung bean seedlings, the content of carbohydrates was also observed to increase under the exposure of DBP (Ma et al., 2014). This study showed that increasing soluble sugar accumulation acts as an early defensive strategy of plants against phthalate stress. Mostly three types of sugars have been documented *i.e.,* disaccharides, refinosis, and fructans to encompass plant stress responses and adaptation (Keunen et al., 2013). Therefore, the elevation in the content of soluble sugars in the present work might be a defense strategy of barley plants against DBP stress.

The plants treated with DBP showed a significant decline in the content of proteins after 15, 30, and 60 days of stress. After 120 days of exposure, the trend was parabolic means it increased initially and then decreased. In a study, Bok choy under DBP stress showed an enhancement in the levels of some proteins, while some other proteins were decreased or even disappeared (Liao, Yen & Wang, 2009). The content of soluble proteins decreased in cucumber seedlings under the treatment of DBP (Wang et al., 2016). The DBP stress to *Lettuce sativus* for 28 days was recorded to decline the content of protein (Gao et al., 2020). Thus, an increase in protein content reflected more sensitivity towards phthalate stress and might have enhanced the expression of stress proteins in 120 days treated plant samples. Additionally, DBP might have boosted the expression of genes involved in the production of heat shock proteins and other stress-related proteins. On the other hand, the decrease in protein content might be due to the degradation and down-regulation of different genes.

In the present study, a significant increase in the content of proline was observed in DBP treated plants. In a similar study, a significant increase in the proline content of water celery was found under BBP exposure at higher doses (Chen et al., 2011). In cucumber seedlings, there was an increase in the proline content was recorded in dose- and time-dependent manner under DBP stress (Zhang et al., 2015). The rise in proline content is intended primarily for water potential homeostasis, which is a crucial component in maintaining normal cell activity and plant metabolism (Wani et al. 2017). Aside from being a compatible solute, proline maintains membrane structure, protein conformation, and scavenges ROS (Szabados & Savouré, 2010). Hence, the exposure of DBP in this study may have prompted the accumulation of proline by activating key transcription pathways.

The content of MDA increased in barley plants for 15 and 30 days, whereas there was an overall decreasing trend in the content of MDA after 60 and 120 days of exposure. In a study, the content of MDA in the duckweed membrane system was shown to be affected by DBP exposure (Huang et al., 2006). Whereas, in the study of Li et al. (2006), DBP exposure to *V. spiralis, P. maackianus* declined MDA content. In rape seedlings, the content of MDA under DBP treatment was recorded to be stimulated (Zhang et al., 2015). Thus, variations in MDA content are considered as an indication of oxidative damage that might be due to the overproduction of ROS in DBP stressed plants.

In the present study, there was an increase in the content of H_2_O_2_ in barley plants treated with DBP for 15–120 days. The *in-situ* aggregation of H_2_O_2_ in *Spirodela polyrhiza* was observed after 3 days of exposure to DEP (Cheng, 2012). The H_2_O_2_ content has been reported to increase with the increase in doses of phthalate and exposure time in cucumber seedlings (Zhang et al., 2015). H_2_O_2_ content was found to increase with DMP stress of 100 mg/L to *Cucumis sativus* L. (Zhang et al., 2016). A substantial accumulation of H_2_O_2_ was found in DEHP exposed wheat plants at the jointing and booting phases with a rise in concentrations (Gao et al., 2018). Under stressed conditions, an increased level of H_2_O_2_ acts as a signal that triggers stress acclimation in the plant.

Stressed plants are reported to show an increase in ROS level, thereby to preserve redox homeostasis, the gridded antioxidant defense system gets activated *via* the regulation of specific antioxidant enzymes. In this work, SOD activity was found to increase significantly in DBP stressed plants for 15, 30, and 60 days. After 120 days of exposure, SOD activity was found to increase only at 2400 ppm. SOD serves as a first-line defense by catalyzing the superoxide radical *via* dismutation reaction (Winterbourn, 2020). The activity of SOD was reported to increase in the shoots and roots of mung seedlings exposed to 5, 20, 100 mg/kg DBP for 72 h, while a decline was observed at 500 mg/kg DBP as compared to control (Ma et al., 2014). The cucumber seedlings under DBP stress showed dose- and time-dependent increases in SOD activity. On contrary, in the study of Cheng & Cheng (2012), it was documented that the exposure of DEP declined SOD activity in *S. polyrhiza* after 7 days of stress that was supposed to occur due to down-regulation of four SOD isoenzymes. The decreased SOD activity of rape seedlings was reported to reflect the high adaptability of the plant under DBP stress (Ma et al., 2013b). Furthermore, in another study, SOD activity was reported to decline by 47% at higher concentrations of DBP after 7 days of exposure (Zhang et al., 2015). In the present study, the effects of DBP on the SOD activity of barley plants may perhaps be due to the enhanced ROS generation as well as due to the down-regulation of SOD isoenzymes under prolonged DBP stress. The SOD mediated dismutation reaction is reported to be associated with the accumulation of H_2_O_2_ that can range from 1 to 15 µM under stressed conditions (Corpas, Gupta & Palma, 2015). H_2_O_2_ is not so toxic nevertheless can form ROS like the hydroxide ion (OH^−^) (*i.e.,* more damaging) through the Haber-Weiss reaction (Czarnocka & Karpiński, 2018). Therefore, there is a consecutive defense mechanism that involves the depletion of H_2_O_2_ *via* the action of POD, CAT, and APX enzymes before it can enter other reactions. The barley plants treated with DBP caused the overall increase in the activity of POD in all exposure durations. The results of the present study are comprehensible with other reports. The activity of POD was observed to increase significantly in cucumber seedlings under the exposure of dimethyl phthalate (DMP) for 7 days (Zhang et al., 2016). Peroxidases are grouped as class III peroxydases that are the only antioxidative enzymes to scavenge H_2_O_2_ in the apoplast of the cell (Welinder, 1992). Consequently, the stimulated POD activity under DBP stress maybe for the foraging of H_2_O_2_ accumulated in the apoplast of the plant cells. There was an apparent increase in CAT activity in barley plants under DBP stress for 15 and 30 days of treatment. CAT activity was found to be either increased or decreased in comparison to control after 60 and 120 days of treatment with DBP. In several studies, the increase in CAT activity is observed to be positively correlated with H_2_O_2_ generation during stressed conditions. Moreover, phthalates are also reported to enhance the production of H_2_O_2_ remarkably in plants (Bai et al., 2009). CAT is an enzyme that scavenges H_2_O_2_ produced in peroxisomes under stressed conditions. In the present study, the enhanced activity of CAT is attributed to its indispensable role in the detoxification of H_2_O_2_ accumulated under phthalates stress in plants. Thus, the accumulation of H_2_O_2_ might be because of photorespiratory oxidation or *β*-oxidation induced by the exposure of DBP. In this work, APX activity followed the overall increasing trend under the treatment of DBP for all exposure periods. Ma et al. (2014) observed similar results under the exposure of DEHP in the shoots and roots of mung bean seedlings. In wheat plants under DEHP exposure, the activity of APX was observed to increase as compared to control (Gao et al., 2018). Wheat plants also showed aggregation in APX activity under DEHP and DBP stress for 14, 24, and 40 days (Gao et al. 2019). APX catalyzes the reduction of H_2_O_2_ using ascorbate as an electron donor. Therefore, it plays an important role in the mitigation of intracellular ROS levels (Abbas et al., 2018). The activity of GR showed increasing effects after the treatment of DBP for 15–120 days. The activity of GR was also observed to increase under the exposure of DEP in *S. polyrhiza* (Cheng & Cheng, 2012). The activity of GR was observed to be altered significantly under the exposure of phthalates (Kumari & Kaur, 2020a). The other pollutants are also reported to be aggravated the GR activity in different crop plants (Katerova et al., 2021).

Besides, the PCA and Pearson’s correlation analyses supported the finding of accumulation and morpho-physiological investigations. In PCA analysis, the positive loadings depicted that all the variables are positively interrelated and have a crucial role in the maintenance of the plant’s homeostasis under the stressed environment (Sharma & Kaur, 2019). These statistical tools also revealed that the main cause behind the various consequences of DBP accumulation and translocation. The similar observations were found in the study of Sakouhi et al. (2021).

FTIR is a well-known technique for macromolecular analysis because the chemical bonds of macromolecules have different vibrational characteristics. Thus, the spectra can provide information regarding biochemical constituents of plants like carbohydrates, proteins, lipids, *etc*. In the control sample, the IR absorption band was observed at 3395 cm^−1^ which indicated the symmetric stretch of the aliphatic primary amine group (–NH) which confirmed the presence of proteins. The peak that emerged at 2920 cm^−1^ is related to asymmetric vibration of C-H bonds of an aliphatic group (=CH_2_) of the fatty acid backbone (Driver et al., 2015; Xue et al., 2018). The peaks between 2000–2500 cm^−1^ are referred as a triple bond region. Therefore, the peak at 2350 cm^−1^ indicated C ≡C group and peaks found at 1735, 1718, 1654, and 1638 cm^−1^ indicated the carbonyl group (–C =O) which showed the presence of lipids (Lahlali et al., 2014; Driver et al., 2015). The peak at 1246 cm^−1^ corresponds to C-O stretching vibration of esters of *α* and *β*-unsaturated acids (Coates, 2006). The vibrational peaks found between wavenumber 900–1200 cm^−1^ is for polysaccharides (Driver et al., 2015). The absorption bands between 675–950 cm^−1^ are attributed to aromatic C–H out-of-plane bend. DBP treated samples for 60 days showed the shifting of existing peaks as well as the disappearance of some new peaks which reflected the adverse effects of DBP on cellular proteins, carbohydrates, and lipids as compared to control. Thus, the peaks in the treated plant sample were not merged and had less intensity, indicating a lower protein and carbohydrate content under DBP-stressed plants (D’Souza et al., 2008). Also, these results are coherent with the findings of Sharma & Kaur (2019).

### Proposed mechanism

In the current study, there was significant accumulation and translocation of DBP was recorded in roots, shoots, and seeds of barley plants. Based on statistical analyses, the association of morpho-physiological parameters with DBP accumulation and translocation was also verified. Thus, the key cue behind phytotoxic responses induced by DBP was its accumulation and translocation that have incremented the ROS generation in barley. The aggravation in ROS level disturbed cellular homeostasis and wielded oxidative stress (Sharma & Kaur, 2019; Gao et al., 2021). The oxidative stress exerted several physiological and metabolic perturbations in barley plants. On the other hand, to cope up with DBP stress, plants have activated their antioxidative defense system (Kumari & Kaur, 2020b). Based on this study’s outcomes, it is revealed that the accumulation of DBP in different plant parts hindered the normal growth and metabolism that ultimately caused a reduction in crop yield. Moreover, the accumulation of DBP also raises food security risks.

## Conclusions

Widespread use of phthalates-containing products in everyday life has caused major problems, even though they are abundant in arable soils, raising global food security worries. The above-recorded observations in barley under DBP exposure indicated that it has significantly affected all the indices associated with the normal growth and physiological state of the plant *via* its considerable accumulation and translocation. The impact of DBP on barley was recorded to be increased with its doses and exposure periods. The maximal impact was observed in 60 days DBP stressed plants, while the pattern of DBP effects on barley after 120 days deviate as the normal phenomenon of seed setting might have occurred in the untreated plants that later led to senescence as a normal part of plant development. Moreover, the outcomes of this work are well explained as well as they are coherent with several studies based on phthalates as well as other organic pollutants’ exposure to cereal crops. Thus, the results of the present study provided a basis for the development of edible or genetically resistant plants *via* using the techniques of plant breeding and protected horticulture.

##  Supplemental Information

10.7717/peerj.12859/supp-1 Supplemental Information 1 Physico-chemical analysis of soil used in the field study

10.7717/peerj.12859/supp-2 Supplemental Information 2 ANOVA summary for biochemical parameters and activities of antioxidative enzymes **means significant at *p* ≤ 0.01, *p ≤ 0.05

10.7717/peerj.12859/supp-3 Supplemental Information 3 Pearson’s correlation matrix for different parameter of barley plants exposed to DBP for 15, 30, 60, and 120 days **. Correlation is significant at the 0.01 level (2-tailed). *. Correlation is significant at the 0.05 level (2-tailed). Here, DW: dry weight; NPP: net primary productivity; TChl: total chlorophyll; CRT: carotenoids; CRB: carbohydrates; PRT: proteins; PRL: proline; MDA: malonaldehyde; H_2_O_2_: hydrogen peroxide; SOD: superoxide dismutase; POD: guaiacol peroxidase; CAT: catalase; APX: ascorbate peroxidase; SAF: shoot accumulation factor; RAF: root accumulation factor; TF: translocation factor; SW: seed weight; SN: seed number.

10.7717/peerj.12859/supp-4 Supplemental Information 4 Dataset for Fig. 2

10.7717/peerj.12859/supp-5 Supplemental Information 5 Dataset for Fig. 2

10.7717/peerj.12859/supp-6 Supplemental Information 6 Dataset for Table 1

## Additional Information and Declarations

### Data Availability

The following information was supplied regarding data availability: The raw data are available in the Supplementary Files.
