# Supplementary material for: Uptake of a plasticizer (di-n-butyl phthalate) impacts the biochemical and physiological responses of barley
Source: PeerJ. 2022 Feb 14;10:e12859. doi: 10.7717/peerj.12859 (PMC8852270; doi:10.7717/peerj.12859)
Supplement: Supplemental Information 1 [file peerj-10-12859-s001.docx]

| Parameters | Observations | |
| --- | --- | --- |
| Physical characteristics | | |
| pH | 7.9±0.05 | |
| Electric conductivity (dS m^-1^) | 615.00±30.69 | |
| Soil texture | 81.97% (sand) | Sandy loam |
|  | 14.00% (clay) |  |
|  | 4.03% (slit) |  |
| Chemical characteristics | | |
| Total organic carbon (g/kg) | 3.75±0.32 | |
| TKN (mg/g soil) | 11.55±1.07 | |
| TAP (mg/g soil) | 0.166±0.007 | |
| Na (mg/g soil) | 0.893±0.013 | |
| K (mg/g soil) | 0.546±0.016 | |
| Zn (mg/g soil) | 0.039±0.004 | |
| Cu (mg/g soil) | 0.007±0.001 | |
| Co (mg/g soil) | 0.003±0.001 | |
| Mn (mg/g soil) | 0.182±0.002 | |
