# Supplementary material for: Uptake of a plasticizer (di-n-butyl phthalate) impacts the biochemical and physiological responses of barley
Source: PeerJ. 2022 Feb 14;10:e12859. doi: 10.7717/peerj.12859 (PMC8852270; doi:10.7717/peerj.12859)
Supplement: Supplemental Information 2 — **means significant at p ≤ 0.01, *p ≤ 0.05 [file peerj-10-12859-s002.docx]

| **Total Chl content** | | | | | |
| --- | --- | --- | --- | --- | --- |
| **Source of error** | **SS** | **df** | **MSS** | **F-ratio** | **HSD** |
| **Treatment (T)** | 87.98 | 3 | 29.33 | 8.48^**^ | 3.06 |
| **Dose (D)** | 139.25 | 3 | 46.42 | 13.42^**^ |  |
| **T × D** | 189.83 | 9 | 21.09 | 6.10^**^ |  |
| **Error** | 442.70 | 128 | 3.46 |  |  |
| **Total** | 859.77 | 143 |  |  |  |
| **Carotenoids content** | | | | | |
| **Treatment (T)** | 6.06 | 3 | 2.02 | 19.11^**^ | 0.54 |
| **Dose (D)** | 18.11 | 3 | 6.04 | 57.10^**^ |  |
| **T × D** | 14.72 | 9 | 1.64 | 15.47^**^ |  |
| **Error** | 13.54 | 128 | 0.11 |  |  |
| **Total** | 52.43 | 143 |  |  |  |
| **Carbohydrate content** | | | | | |
| **Treatment (T)** | 436673.02 | 3 | 145557.67 | 257.50^**^ | 39.15 |
| **Dose (D)** | 7310.52 | 3 | 2436.84 | 4.31^**^ |  |
| **T × D** | 13674.60 | 9 | 1519.40 | 2.69^**^ |  |
| **Error** | 72355.31 | 128 | 565.28 |  |  |
| **Total** | 530013.44 | 143 |  |  |  |
| **Protein content** | | | | | |
| **Treatment (T)** | 0.50 | 3 | 0.17 | 18.42^**^ | 0.157 |
| **Dose (D)** | 0.70 | 3 | 0.23 | 25.64^**^ |  |
| **T × D** | 1.27 | 9 | 0.14 | 15.46^**^ |  |
| **Error** | 1.17 | 128 | 0.01 |  |  |
| **Total** | 3.63 | 143 |  |  |  |
| **Proline content** | | | | | |
| **Treatment (T)** | 26877.57 | 3 | 8959.19 | 59.82^**^ | 20.15 |
| **Dose (D)** | 12721.44 | 3 | 4240.48 | 28.31^**^ |  |
| **T × D** | 9731.42 | 9 | 1081.27 | 7.22^**^ |  |
| **Error** | 19169.47 | 128 | 149.76 |  |  |
| **Total** | 68499.90 | 143 |  |  |  |
| **MDA content** | | | | | |
| **Treatment (T)** | 237343.67 | 3 | 79114.56 | 107.44^**^ | 44.68 |
| **Dose (D)** | 2526.19 | 3 | 842.06 | 1.14 |  |
| **T × D** | 29147.77 | 9 | 3238.64 | 4.40^**^ |  |
| **Error** | 94249.76 | 128 | 736.33 |  |  |
| **Total** | 363267.38 | 143 |  |  |  |
| **H_2_O_2_ content** | | | | | |
| **Treatment (T)** | 4304.46 | 3 | 1434.82 | 133.92^**^ | 5.39 |
| **Dose (D)** | 5170.92 | 3 | 1723.64 | 160.88^**^ |  |
| **T × D** | 4083.56 | 9 | 453.73 | 42.35^**^ |  |
| **Error** | 1371.34 | 128 | 10.71 |  |  |
| **Total** | 14930.28 | 143 |  |  |  |
| **SOD activity** | | | | | |
| **Treatment (T)** | 294638.72 | 3 | 98212.91 | 29.37^**^ | 95.23 |
| **Dose (D)** | 121640.27 | 3 | 40546.76 | 12.12^**^ |  |
| **T × D** | 127332.16 | 9 | 14148.02 | 4.23^**^ |  |
| **Error** | 428059.57 | 128 | 3344.22 |  |  |
| **Total** | 971670.72 | 143 |  |  |  |
| **POD activity** | | | | | |
| **Treatment (T)** | 0.062 | 3 | 0.0206 | 29.68^**^ | 0.0434 |
| **Dose (D)** | 0.034 | 3 | 0.0113 | 16.21^**^ |  |
| **T × D** | 0.033 | 9 | 0.0037 | 5.34^**^ |  |
| **Error** | 0.089 | 128 | 0.0007 |  |  |
| **Total** | 0.218 | 143 |  |  |  |
| **CAT activity** | | | | | |
| **Treatment (T)** | 0.014 | 3 | 0.0047 | 17.72^**^ | 0.0269 |
| **Dose (D)** | 0.005 | 3 | 0.0017 | 6.21^**^ |  |
| **T × D** | 0.007 | 9 | 0.0008 | 3.08^**^ |  |
| **Error** | 0.034 | 128 | 0.0003 |  |  |
| **Total** | 0.061 | 143 |  |  |  |
| **APX activity** | | | | | |
| **Treatment (T)** | 31.01 | 3 | 10.34 | 36.06^**^ | 0.88 |
| **Dose (D)** | 7.53 | 3 | 2.51 | 8.76^**^ |  |
| **T × D** | 8.84 | 9 | 0.98 | 3.43^**^ |  |
| **Error** | 36.69 | 128 | 0.29 |  |  |
| **Total** | 84.07 | 143 |  |  |  |
| **GR activity** | | | | | |
| **Treatment (T)** | 0.032 | 3 | 0.0107 | 12.01^**^ | 0.0492 |
| **Dose (D)** | 0.030 | 3 | 0.0100 | 11.19^**^ |  |
| **T × D** | 0.018 | 9 | 0.0020 | 2.24^*^ |  |
| **Error** | 0.114 | 128 | 0.0009 |  |  |
| **Total** | 0.195 | 143 |  |  |  |

^**^means significant at p≤0.01, ^*^p≤0.05
