# Supplementary material for: Uptake of a plasticizer (di-n-butyl phthalate) impacts the biochemical and physiological responses of barley
Source: PeerJ. 2022 Feb 14;10:e12859. doi: 10.7717/peerj.12859 (PMC8852270; doi:10.7717/peerj.12859)
Supplement: Supplemental Information 3 — **. Correlation is significant at the 0.01 level (2-tailed). *. Correlation is significant at the 0.05 level (2-tailed). Here, DW: dry weight; NPP: net primary productivity; TChl: total chlorophyll; CRT: carotenoids; CRB: carbohydrates; PRT: proteins; PRL: proline; MDA: malonaldehyde; H2O2: hydrogen peroxide; SOD: superoxide dismutase; POD: guaiacol peroxidase; CAT: catalase; APX: ascorbate peroxidase; SAF: shoot accumulation factor; RAF: root accumulation factor; TF: translocation factor; SW: seed weight; SN: seed number. [file peerj-10-12859-s003.docx]

| **Correlations matrix** | | | | | | | | | | | | | | | | | | | |
| --- | --- | --- | --- | --- | --- | --- | --- | --- | --- | --- | --- | --- | --- | --- | --- | --- | --- | --- | --- |
| **Parameters** | **DW** | **NPP** | **TChl** | **CRT** | **CRB** | **PRT** | **PRL** | **MDA** | **H_2_O_2_** | **SOD** | **POD** | **CAT** | **APX** | **GR** | **RAF** | **SAF** | **TF** | **SW** | **SN** |
| **DW** | 1 |  |  |  |  |  |  |  |  |  |  |  |  |  |  |  |  |  |  |
| **NPP** | .929^**^ | 1 |  |  |  |  |  |  |  |  |  |  |  |  |  |  |  |  |  |
| **TChl** | -.210 | .028 | 1 |  |  |  |  |  |  |  |  |  |  |  |  |  |  |  |  |
| **CRT** | .069 | .307 | .688^**^ | 1 |  |  |  |  |  |  |  |  |  |  |  |  |  |  |  |
| **CRB** | .599^*^ | .416 | -.352 | -.027 | 1 |  |  |  |  |  |  |  |  |  |  |  |  |  |  |
| **PRT** | -.145 | .006 | .722^**^ | .688^**^ | -.138 | 1 |  |  |  |  |  |  |  |  |  |  |  |  |  |
| **PRL** | .001 | -.166 | -.378 | -.229 | .676^**^ | -.316 | 1 |  |  |  |  |  |  |  |  |  |  |  |  |
| **MDA** | .637^**^ | .454 | -.320 | -.156 | .839^**^ | -.122 | .627^**^ | 1 |  |  |  |  |  |  |  |  |  |  |  |
| **H_2_O_2_** | -.258 | -.401 | -.246 | -.202 | .471 | -.006 | .664^**^ | .415 | 1 |  |  |  |  |  |  |  |  |  |  |
| **SOD** | -.170 | -.188 | -.401 | -.303 | -.303 | -.654^**^ | .012 | -.377 | -.208 | 1 |  |  |  |  |  |  |  |  |  |
| **POD** | .259 | .064 | -.583^*^ | -.582^*^ | .444 | -.737^**^ | .675^**^ | .436 | .156 | .430 | 1 |  |  |  |  |  |  |  |  |
| **CAT** | .188 | .028 | -.620^*^ | -.446 | .257 | -.786^**^ | .514^*^ | .182 | .037 | .680^**^ | .890^**^ | 1 |  |  |  |  |  |  |  |
| **APX** | .525^*^ | .309 | -.632^**^ | -.361 | .886^**^ | -.462 | .722^**^ | .729^**^ | .381 | -.038 | .732^**^ | .549^*^ | 1 |  |  |  |  |  |  |
| **GR** | .231 | .010 | -.806^**^ | -.564^*^ | .433 | -.816^**^ | .550^*^ | .303 | .233 | .542^*^ | .804^**^ | .863^**^ | .711^**^ | 1 |  |  |  |  |  |
| **RAF** | -.255 | -.264 | -.180 | -.014 | -.019 | .041 | -.105 | -.165 | .179 | -.160 | -.366 | -.276 | -.038 | -.052 | 1 |  |  |  |  |
| **SAF** | -.234 | -.267 | -.260 | -.191 | -.101 | .069 | -.180 | -.227 | .156 | -.094 | -.315 | -.250 | -.086 | -.160 | .354 | 1 |  |  |  |
| **TF** | -.243 | -.297 | -.215 | -.285 | -.083 | .035 | -.113 | -.184 | .175 | -.088 | -.211 | -.219 | -.052 | -.161 | .186 | .963^**^ | 1 |  |  |
| **SW** | .770^**^ | .533^*^ | -.334 | -.110 | .738^**^ | -.192 | .377 | .804^**^ | .197 | -.126 | .445 | .372 | .621^*^ | .425 | -.323 | -.271 | -.242 | 1 |  |
| **SN** | .838^**^ | .605^*^ | -.388 | -.147 | .730^**^ | -.250 | .327 | .783^**^ | .077 | -.091 | .486 | .410 | .661^**^ | .462 | -.318 | -.262 | -.237 | .986^**^ | 1 |

**. Correlation is significant at the 0.01 level (2-tailed).

*. Correlation is significant at the 0.05 level (2-tailed).

Here, DW: dry weight; NPP: net primary productivity; TChl: total chlorophyll; CRT: carotenoids; CRB: carbohydrates; PRT: proteins; PRL: proline; MDA: malonaldehyde; H_2_O_2_: hydrogen peroxide; SOD: superoxide dismutase; POD: guaiacol peroxidase; CAT: catalase; APX: ascorbate peroxidase; SAF: shoot accumulation factor; RAF: root accumulation factor; TF: translocation factor; SW: seed weight; SN: seed number.
